# Role of Oral Veillonella Species in Predicting Surgical Site Infections After Maxillofacial Trauma: A Prospective Observational Study

**DOI:** 10.7759/cureus.66158

**Published:** 2024-08-05

**Authors:** Mahima Seetaram, Vivek N, Abinaya Subramanian, Anusha Gopinathan, Leela KV, Saravanan Chandran, Magesh K T, Karthik Ramakrishnan

**Affiliations:** 1 Department of Oral and Maxillofacial Surgery, Sri Ramaswamy Memorial (SRM) Kattankulathur Dental College and Hospital, SRM Institute of Science and Technology (SRMIST), Chennai, IND; 2 Department of Microbiology, Sri Ramaswamy Memorial (SRM) Medical College Hospital and Research Centre, SRM Institute of Science and Technology (SRMIST), Chennai, IND; 3 Department of Oral Pathology and Microbiology, Sri Ramaswamy Memorial (SRM) Kattankulathur Dental College and Hospital, SRM Institute of Science and Technology (SRMIST), Chennai, IND

**Keywords:** osteomyelitis, prevention, maxillofacial fractures, surgical site infection (ssi), veillonella

## Abstract

Introduction: There are comparatively fewer surgical site infections after craniofacial trauma than after trauma to the extremities, and the etiology is complex. Gram-negative facultative anaerobic bacteria *Veillonella* is a common commensal in the oral cavity and has been linked to osteomyelitis and surgical site infections in prosthetic joint infections. They serve as early biological indicators.

Aims/objectives: This study aims to assess the presence of *Veillonella* in patients presenting with maxillofacial trauma, to document the difference in colony count in patients requiring surgical intervention at different time intervals as against patients with surgical site infections, and to provide better hospital care and management so as to improve the standard of care with an attempt to prevent the possibility of postoperative surgical site infections.

Methodology: In this study, individuals with trauma/fractures of the maxillofacial region requiring surgical intervention at varied time spans, early, intermediate, and late, were included. After obtaining informed consent, the examination was done; the fracture type and site were noted, and a swab was taken on the day of admission, on the day of surgery, and on the day of discharge and given for microbiological evaluation. Findings were recorded.

Results: The primary and secondary objectives of the study were established. The mean colony count in colony-forming units/milliliter for patients undergoing early surgical intervention, on the day of admission, was 2.01E+0.6. On the day of discharge, the mean colony count was 1.51E+0.6. In contrast, for patients with surgical site infection, on the day of admission, the mean was 6.5E+0.7, and on the day of discharge, the mean colony count reduced to 4.01E+0.6. The time-colony-forming unit graph showed a difference in the colony count of *Veillonella* in patients operated at different time intervals as against patients with surgical site infection and modified relation with a number of other oral commensals. The colony count in patients with osteomyelitis was found and compared.

Conclusion: There is a change in the colony count of *Veillonella* species and its relation to their commensals when intervened at different time intervals. Our study indicates that the estimation of *Veillonella* species and the colony count could aid in determining the possibility of a surgical site infection. This study also stresses on the appropriate reporting of maxillofacial trauma in cases of a poly-trauma for appropriate management.

## Introduction

Multiple bacterial species are present in the oral biofilm as a result of the complex interaction of salivary glycoproteins that have been adsorbed on the surface of enamel [1]. As against random simultaneous colonization, these biofilms are created via sequential, reproducible, and selective colonization [1,2].

Saliva is considered to comprise of biological markers with important contribution to composition of the oral microbiota [3,4]. Furthermore, saliva is important in oral biofilm development and maintenance [4].

*Veillonella* species are present as commensal organisms in the gastrointestinal, genitourinary, and respiratory systems of humans and animals [5-7]. *Veillonella* subtypes are most commonly involved in dental caries and poor periodontal conditions. They are indicted in meningitis, endocarditis, bacteremia, discitis, vertebral osteomyelitis, and prosthetic joint infection [8-12]. These species have been isolated from lesions, osteomyelitis, secondary septicemia, and prosthetic joint infections [11]. They act as sensitive indicators and bridges for polymicrobial infections and early representatives of excessive acid production [13-16].

*Veillonella* is less likely to cause infection in isolation and is associated more commonly with open fractures. Hence, an estimate of these species would help to probably predict the future course of healing or possible occurrence of sepsis post the reduction and fixation of fractures.

This study was presented as a poster for the 46th National Conference of AOMSI - 2022 in Indore, India.

## Materials and methods

This prospective observational study was conducted over a period of 12 months, in the dental college with the department of microbiology. This study was approved by Sri Ramaswamy Memorial (SRM) Medical College Hospital Institutional Ethics Committee (SRMIEC) dated 11/10/2022, the approval number being SRMIEC-ST0922-59.

Patients with a history of trauma leading to untreated fractures of the maxillofacial region, within the age group of 18-70 years, were included. Patients with systemic comorbidities, with ASA III or ASA IV (American Society of Anesthesiologists Physical Status Classification), and on antibiotic therapy were excluded from our study. The purpose of this study was explained, and written informed consent was taken. The principal investigator recorded the general details of the participants. For all the participants, the socioeconomic status and background were matched.

Due to the type of trauma and multidisciplinary approach based on complications of trauma, the time of fracture, and the time of surgical intervention, the patients were divided into three groups: early (0-2 days), intermediate (3-5 days), and late surgical intervention (6 days and above).

In each group, three unstimulated saliva samples and swabs of patients at three different intervals, on the day of admission, on the day of surgical intervention, and on the day of discharge, were collected. The group included the collection of samples of subjects with different time intervals, early, intermediate, and late, and also samples of patients presenting with osteomyelitis and surgical site infections (SSI). Examination and sample collection was done by a single examiner. The microbiological examination was done by an experienced microbiologist.

Sample collection

Patients were refrained from following any oral hygiene practice, consumption of food, and associated habits for at least 6-8 hours prior to sample collection. The general oral hygiene status of patients was recorded and medications, if administered, were noted. A swab was taken from the site of the fracture, and then patients were asked to sit still for 2-3 minutes. Unstimulated saliva was collected in three separate vials per patient, on the day of admission, on the day of surgery, and on the day of discharge. Then the samples were sent to the department of microbiology for culture study. 

Culture conditions

**Figure 1:**
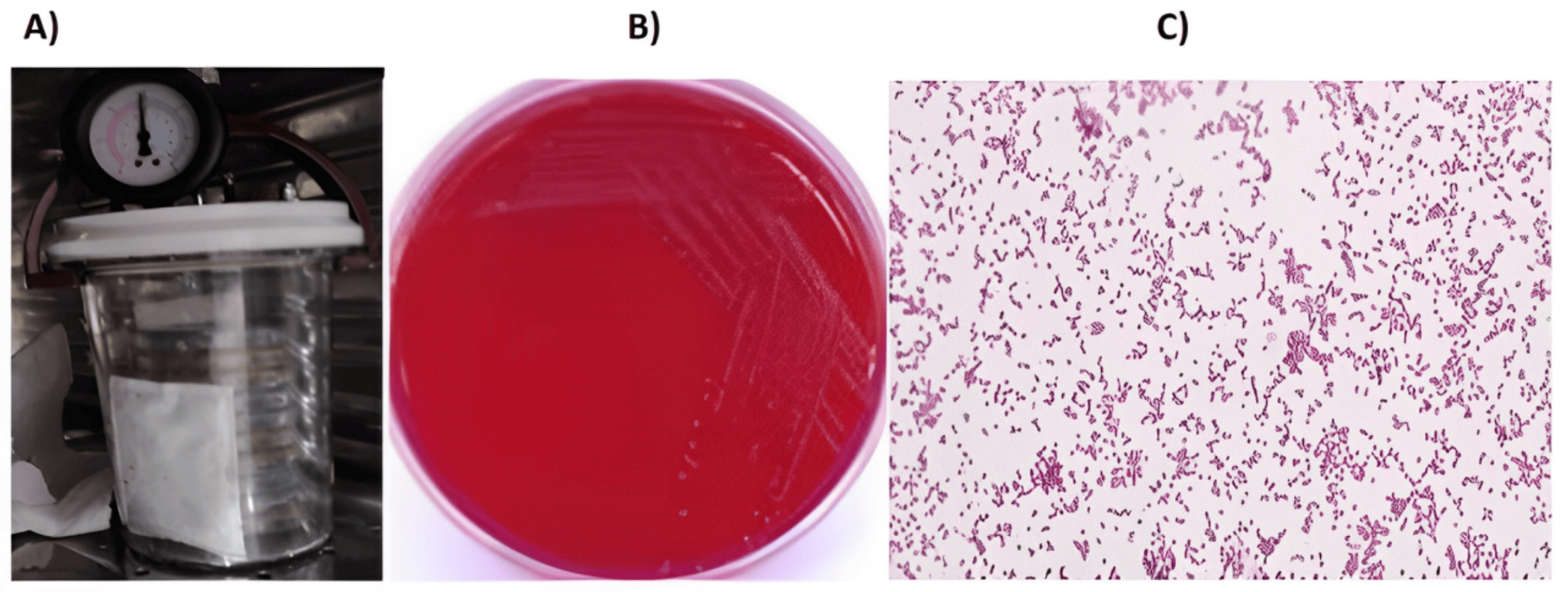
From the left to the right: (A) McIntosh and Fildes' jar, (B) culture plate, and (C) microscopic view

Saliva samples were diluted with sterile saline, inoculated into brain heart infusion (BHI) (Difco Laboratories, Detroit, Michigan, United States) and *Veillonella* blood agar, and incubated under anaerobic conditions. *Veillonella* blood agar and BHI had samples incubated for five and seven days, respectively. The bacterial colonies grown on the media were examined and confirmed as gram-negative cocci. VITEK 2 cards (bioMérieux, Marcy-l'Étoile, France) were used to confirm the same (Figure 1).

Tabulation of data

The colony count in colony-forming units/milliliter (CFU/ml) was tabulated on an Excel sheet. The collected data were entered into the Statistical Package for the Social Sciences (SPSS) version 11.5. The results have been expressed as tables and figures. Regression analysis was used to predict the three-microbe culture.

## Results

Demographic

The participants included in the study comprised 11 females and 19 males with an age range of 22-73 years.

Colony numbers

Among the groups, 10 participants comprised those receiving early surgical intervention (0-2 days), nine participants comprised those receiving intermediate surgical intervention (3-5 days), and seven participants comprised those receiving late surgical intervention (>6 days), for whom intraoral samples were collected on the day of admission/takeover, on the day of surgery, and on the day of discharge. For patients with SSI, a sample was also taken on postoperative day 10 in four participants to assess change in colonies, and the following data was obtained. The colony count of *Veillonella* was expressed as CFU/ml.

Early Surgical Intervention

For patients undergoing early surgical intervention, on the day of admission, the mean CFU/ml was 2.01E+0.6 CFU/ml with the range being 2.45E+0.6 CFU/ml and 1.70E+0.6 CFU/ml, the median of which was 1.87E+0.6 CFU/ml. On the day of surgery, the culture revealed a mean of 1.51E+0.6 CFU/ml with a range of 1.64E+0.7 CFU/ml to 1.97E+0.6 CFU/ml, the median being 1.65E+0.6 CFU/ml. On the day of discharge, the mean colony count was 1.51E+0.6 CFU/ml with a range of 1.43E+0.6 CFU/ml to 1.68E+0.6 CFU/ml with a median of 1.43E+0.6 CFU/ml.

Intermediate Surgical Intervention

For patients undergoing intermediate surgical intervention, on the day of admission, the mean CFU/ml was 2.62E+0.7 CFU/ml with the range being 1.56E+0.8 CFU/ml and 3.56E+0.6 CFU/ml, the median of which was 2.45E+0.6 CFU/ml. On the day of surgery, the culture revealed a mean of 2.03E+0.6 CFU/ml with a range of 1.46E+0.7 CFU/ml to 2.76E+0.6 CFU/ml, the median being 1.97E+0.6 CFU/ml. On the day of discharge, the mean colony count was 1.72E+0.6 CFU/ml with a range of 1.35E+0.6 CFU/ml to 2.02E+0.6 CFU/ml with a median of 1.76E+0.6 CFU/ml.

Late Surgical Intervention

For patients undergoing late surgical intervention, on the day of admission, the mean CFU/ml was 3.96E+0.7 CFU/ml with the range being 3.83E+0.6 CFU/ml to 4.35E+0.8 CFU/ml, the median of which was 3.83E+0.6 CFU/ml. On the day of surgery, the culture revealed a mean of 3.05E+0.7 CFU/ml with a range of 2.13E+0.6 CFU/ml to 3.89E+0.6 CFU/ml, the median being 3.12E+0.6 CFU/ml. On the day of discharge, the mean colony count was 2.01E+0.6 CFU/ml with a range of 1.70E+0.6 CFU/ml to 2.76E+0.7 CFU/ml with a median of 1.70E+0.6 CFU/ml.

Patients with SSI

In SSI patients, on the day of admission, the mean CFU/ml was 6.5E+0.7 CFU/ml with the range being 5.12E+0.6 CFU/ml and 7.56E+0.6 CFU/ml, the median of which was 6.82E+0.6 CFU/ml. On the day of surgery, the culture revealed a mean of 5.78E+0.7 CFU/ml with a range of 4.02E+0.7 CFU/ml to 6.98E+0.6 CFU/ml, the median being 6.36E+0.6 CFU/ml. On the day of discharge, the mean colony count was 4.01E+0.6 CFU/ml with a range of 2.97E+0.8 CFU/ml to 4.94E+0.8 CFU/ml with a median of 4.12E+0.6 CFU/ml.

**Figure 2:**
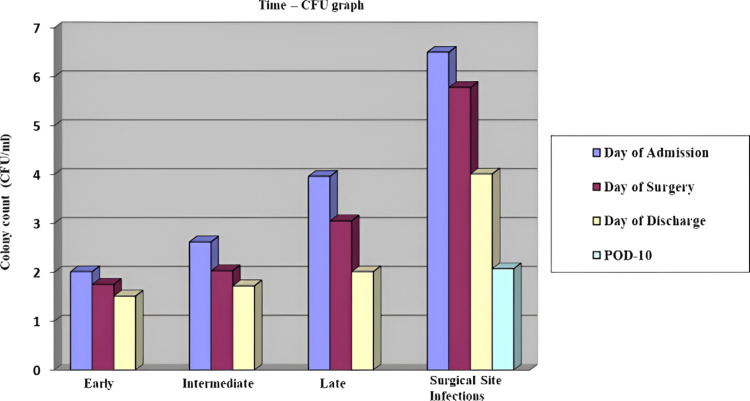
Time of intervention-CFU (mean) graph for Veillonella species CFU: colony-forming units

There was a difference in colony counts based on the time of intervention in the *Veillonella* species (Figure 2).

**Figure 3:**
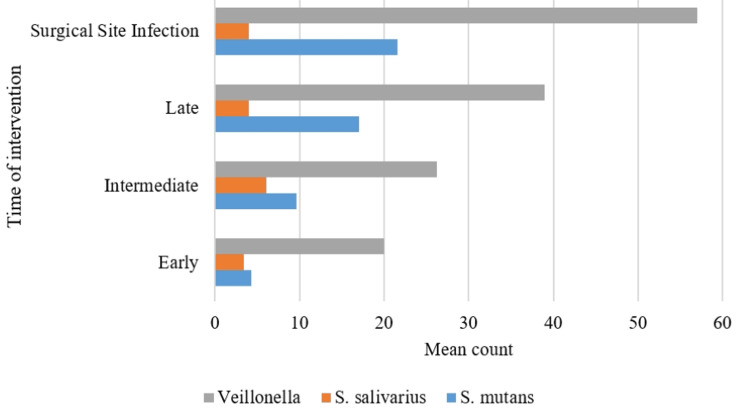
Comparison of Veillonella species, Streptococcus salivarius, and Streptococcus mutans levels

Linear regression analysis shows that *Streptococcus mutans* and *Streptococcus salivarius* colony counts can be significantly predicted. They had a variation in their counts based on the presence and count of *Veillonella* species. As the *Veillonella* count increases, the colony count of *Streptococcus salivarius* decreases (P=0. 013), but that of *Streptococcus mutans* increases (Figure 3).

## Discussion

*Veillonella *spp. are most commonly isolated in the oral cavity; one of the earlier cases of *Veillonella* bacteremia was discussed by Liu et al. and then subsequently by Isner-Horobeti et al. regarding *Veillonella* discitis [3,4].

Though some literature is available for other SSI, the relation to maxillofacial regions is not completely established, even though maxillofacial fractures are majorly directly involved with the oral environment.

Monomicrobial *Veillonella *sp.-detected prosthetic joint infections have been reported and documented wherein treatment with antimicrobial therapy was provided with a two-staged plan combined with six weeks of antibiotic therapy [17]. The primary source of the above-said species was reported to be the oral cavity. Transient bacteremia was observed in patients, which is seeded in various sites including the vertebrae. An article also elaborated on the support provided by the culture as against the just documentation of history [18].

Saliva is widely known as a source of biological markers due to the quick progress made in the field of salivaomics. Saliva is a safe, non-invasive alternative to blood for use in diagnosis and prognosis [19].

A study showed that the growth of *Streptococcus*, a facultative anaerobe, may be inhibited by anaerobic conditions in favor of the growth of *Veillonella*. This could explain the inverse relation and growth rate of various *Streptococcus *when compared with *Veillonella* species [20].

A case was once reported with the radiological appearance of metastatic cancer. However, the pathology and culture data served as important factors in diagnosing underlying infection. Stress was placed on how a failure to accurately diagnose an infection could change the clinical scenario [21].

It can be difficult to diagnose a clinically severe infection brought on by *Veillonella* species. These species are frequently thought of as contaminants and are typically found in polymicrobial processes. They also take a while to become established in society, which might cause a missing or delayed diagnosis. Although they seldom result in life-threatening infections, they have been linked to bacteremia [22].

A case of *Veillonella parvula* vertebral osteomyelitis in an immunocompetent patient was reported. Lack of risk factors with symptomatic presentations revealed disco-vertebral samples as positive for *Veillonella *sp. [23]. 

In our study, the participants with SSI were afebrile but provided clinical presentations like pain and discharge in relation to previously operated sites. The culture study helped in identifying the pathology and confirming the appropriate treatment plan. Our study included participants with a history of trauma requiring surgical intervention based on the time of intervention which was based on factors such as complications, neurosurgical status, combined procedures, etc. The study showed significant variation in the *Veillonella* species count among the three groups when assessed with patients with SSI. The count of *Veillonella* also changed with the presence and absence of *Streptococcus *species.

Though the role of *Veillonella* in trauma, bacteremia, and SSI has been mentioned in the literature, the documented data in maxillofacial SSI is less. This could be considered as a limitation. Another limitation of our study was the lack of homogeneity, as all fractures were included. In the future, a multicentric study with a greater sample size and specific fracture types could be conducted, to further understand the role of these species.

## Conclusions

In this study, the microbiological analysis of swabs and oral rinse from patients with different time spans of surgical intervention and SSI has re-iterated the significance of variation in *Veillonella* colony count in patients with trauma, based on time-CFU graph indicating it to be a possible factor in maxillofacial SSI.

This could also indicate that the estimation of these species and their colony count aids in determining the possibility of SSI. This study also stresses on the appropriate reporting of maxillofacial trauma in cases of a poly-trauma, where the delay in reporting could lead to late intervention, thus increasing the risk of bacteremia. Its further role and interaction with other microbes have also been documented.

Further research on this microorganism could help us understand its interaction with other species and its possible role in the prevention of SSI.
